# Supplementary material for: Towards ubiquitous requirements engineering through recommendations based on context histories
Source: PeerJ Comput Sci. 2022 Jan 3;8:e794. doi: 10.7717/peerj-cs.794 (PMC8771779; doi:10.7717/peerj-cs.794)
Supplement: Supplemental Information 1 [file peerj-cs-08-794-s001.doc]

**
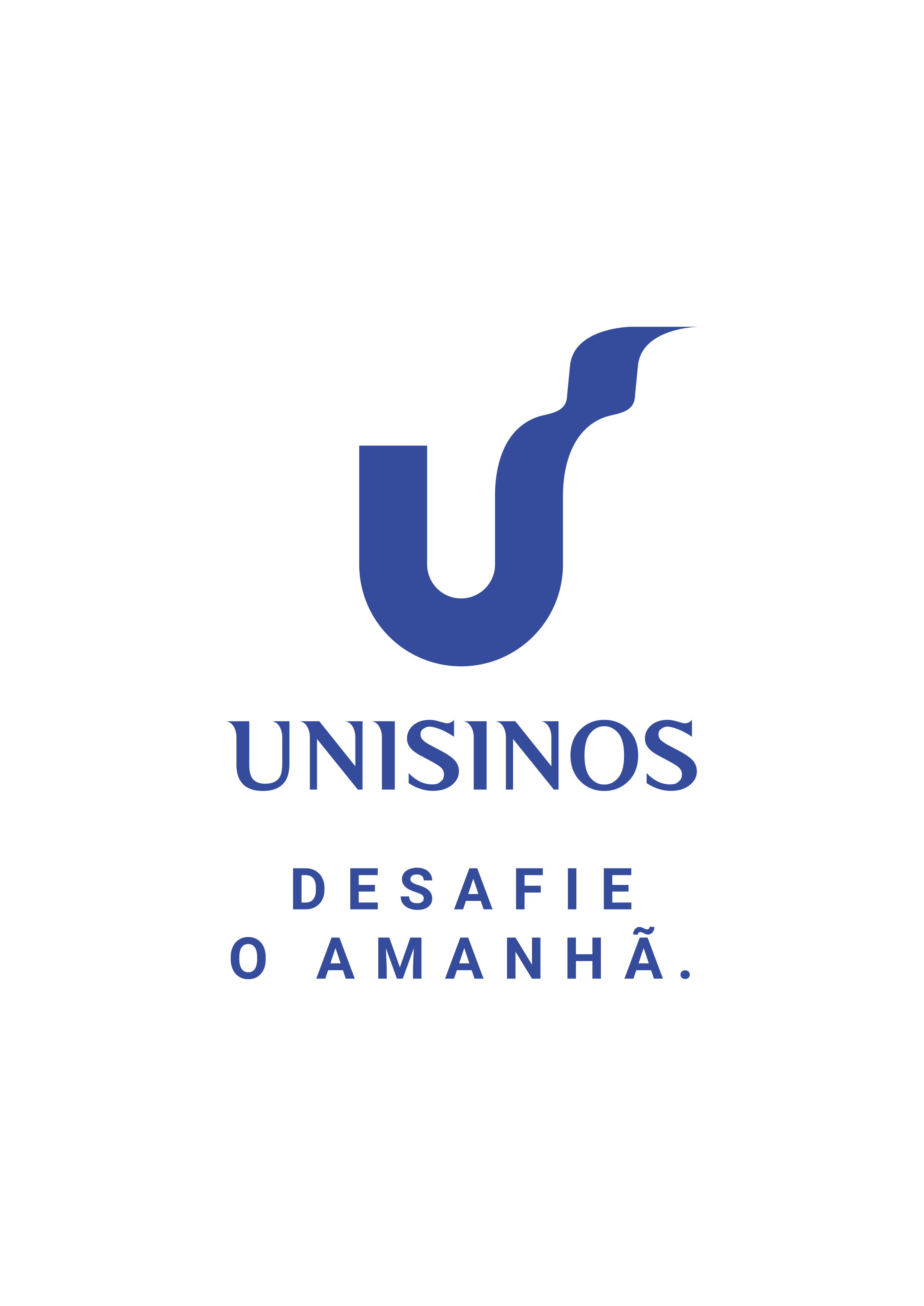
**

***Questionário***

**Nome:**

**E-mail:**

**Data/Hora:**

**1. Qual abordagem sua equipe utiliza atualmente?**

( ) Ágil

( ) Tradicional (PMBoK, RUP, PRINCE2, etc)

( ) Bimodal (modelo híbrido, com características de projetos ágeis e tradicionais)

( ) Não utiliza nenhuma metodologia específica

( ) Outra(s):

**2. Qual sua experiência como Gerente de Projetos ou como membro em equipes de projeto?**

( ) Menos de 2 anos

( ) De 2 a 5 anos

( ) Mais de 5 anos

**3. Qual o tamanho da empresa onde trabalha atualmente?**

( ) Menos de 20 funcionários

( ) De 20 a 100 funcionários

( ) Mais de 100 funcionários

**4. Quais áreas você considera mais críticas para o sucesso do projeto?**

( ) Integração

( ) Escopo

( ) Tempo

( ) Custos

( ) Qualidade

( ) Recursos Humanos

( ) Comunicações

( ) Riscos

( ) Aquisições

( ) Partes Interessadas

**5. Nos projetos em que ocorreram problemas, quais foram as áreas em que os problemas foram identificados?**

( ) Integração

( ) Escopo

( ) Tempo

( ) Custos

( ) Qualidade

( ) Recursos Humanos

( ) Comunicações

( ) Riscos

( ) Aquisições

( ) Partes Interessadas

**6. Quais tipos de sugestão você gostaria de receber de uma ferramenta proativa de gestão de projetos?**

( ) Riscos para o projeto

( ) Alocação de recursos

( ) Possibilidade de atraso

( ) Possibilidade de exceder custos

( ) Sugestão de requisitos e novas funcionalidades

( ) Outro(s):

**7. Você acredita que informações de outros projetos já concluídos poderiam auxiliar na gestão do projeto?**

( ) Sim

( ) Não

( ) Em partes
