# Supplementary material for: Towards ubiquitous requirements engineering through recommendations based on context histories
Source: PeerJ Comput Sci. 2022 Jan 3;8:e794. doi: 10.7717/peerj-cs.794 (PMC8771779; doi:10.7717/peerj-cs.794)
Supplement: Supplemental Information 2 [file peerj-cs-08-794-s002.doc]

**
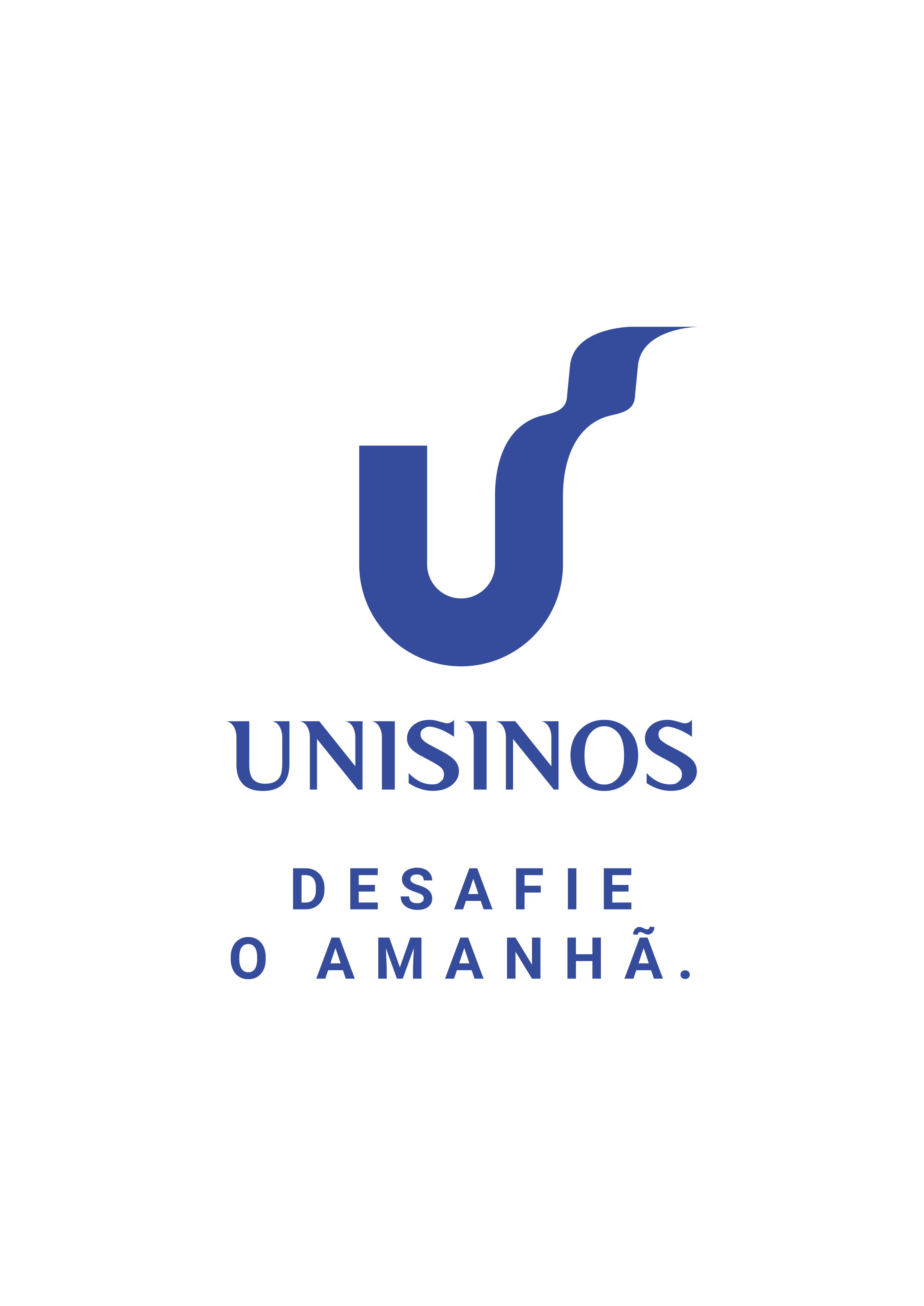
**

***Questionnaire***

**Name:**

**E-mail:**

**Date/Time:**

**1. Which approach does your team use currently?**

( ) Agile

( ) Traditional (PMBoK, RUP, PRINCE2, etc)

( ) Bimodal (hybrid model with characteristics of agile and traditional projects)

( ) The team does not use any specific methodology

( ) Other(s):

**2. What is your experience as a Project Manager or a member of a project team?**

( ) Less than 2 years

( ) From 2 to 5 years

( ) More than 5 years

**3. How many employees work in the company where you work currently?**

( ) Less than 20 employees

( ) From 20 to 100 employees

( ) More than 100 employees

**4. Which areas do you consider most critical to the success of the project?**

( ) Integration

( ) Scope

( ) Time

( ) Costs

( ) Quality

( ) Human Resources

( ) Communications

( ) Risks

( ) Acquisitions

( ) Stakeholders

**5. In the projects where problems occurred, what were the areas in which the problems were identified?**

( ) Integration

( ) Scope

( ) Time

( ) Costs

( ) Quality

( ) Human Resources

( ) Communications

( ) Risks

( ) Acquisitions

( ) Stakeholders

**6. What types of suggestions would you like to receive from a proactive project management tool?**

( ) Risks to the project

( ) Resource allocation

( ) Possibility of delay

( ) Possibility of exceeds costs

( ) Suggestions of requirements and new functionalities

( ) Other(s):

**7. Do you believe that information from other projects already completed could assist in project management?**

( ) Yes

( ) No

( ) Partially
